# Nutritional Support for Liver Diseases

**DOI:** 10.3390/nu15163640

**Published:** 2023-08-19

**Authors:** Dominika Jamioł-Milc, Anna Gudan, Karolina Kaźmierczak-Siedlecka, Joanna Hołowko-Ziółek, Dominika Maciejewska-Markiewicz, Katarzyna Janda-Milczarek, Ewa Stachowska

**Affiliations:** 1 Department of Human Nutrition and Metabolomics, Pomeranian Medical University in Szczecin, 71-460 Szczecin, Poland; 2 Department of Medical Laboratory Diagnostics—Fahrenheit Biobank BBMRI.pl, Medical University of Gdansk, 80-211 Gdansk, Poland

**Keywords:** liver diseases, nutrition, cholangiocarcinoma, NAFLD, NASH, cirrhosis, liver transplantation

## Abstract

The liver is a key organ that is responsible for the metabolism of proteins, fats, and carbohydrates and the absorption and storage of micronutrients. Unfortunately, the prevalence of chronic liver diseases at various stages of advancement in the world population is significant. Due to the physiological function of the liver, its dysfunction can lead to malnutrition and sarcopenia, and the patient’s nutritional status is an important prognostic factor. This review discusses key issues related to the diet therapy of patients with chronic liver diseases, as well as those qualified for liver transplantation and in the postoperative period.

## 1. Introduction

Chronic liver disease (CLD) is the progressive deterioration of liver function lasting more than six months [1]. CLD can be divided into infectious (caused by viral infection- Hepatitis B and C) and non-infectious (i.a. alcoholic liver disease, non-alcoholic fatty liver disease (NAFLD), non-alcoholic steatohepatitis (NASH), autoimmune hepatitis, Wilson disease, and hereditary hemochromatosis). The causes of non-infectious liver diseases include alcoholism, metabolic disorders, toxins, and autoimmune and genetic disorders. Both infectious and non-infectious liver diseases can lead to liver fibrosis and cirrhosis. The stages of the development of chronic liver disease are hepatitis or steatosis or hepatosteatosis, fibrosis, cirrhosis, and hepatocellular carcinoma (HCC) [1].

It is estimated that more than 844 million people worldwide suffer from chronic liver disease, resulting in approximately 2 million deaths per year [2]. The most common cause of chronic liver disease is non-alcoholic fatty liver disease (NAFLD) [3]. It has been estimated that 25% of the world and 90% of the obese population have some degree of NAFLD. By contrast, the prevalence of its active stage, non-alcoholic steatohepatitis (NASH), is around 3–5% [3].

The liver is a key organ in which the numerous metabolic processes of proteins, carbohydrates, and fats take place; therefore, it is responsible for maintaining proper nutritional status. In the advanced stage of the disease, there is a malabsorption of fats, deficiencies in fat-soluble vitamins, a reduction in the level of water-soluble vitamins, and changes in micronutrient metabolism. Thus, the dysfunction of this organ leads to malnutrition and sarcopenia [4]. In patients with liver disease, their diet and nutritional status is an important therapeutic and prognostic factor [5].

In general, patients with chronic liver disease are advised to eat a healthy diet that is varied in foodstuff, devoid of ultra-processed industrialized food, sugar-sweetened beverages, and high-fat food [6]. Recommendations regarding the intake of both macro- and micronutrients are dictated by supplementing the deficits in these ingredients, resulting from the specificity of the disease (absorption disorders, metabolic disorders, e.g., impairment of carbohydrate, protein, and lipid metabolism) or the support of liver function (e.g., the improvement of liver enzymes) [5].

Alcohol should definitely be excluded because ethanol and/or its metabolites cause metabolic, biochemical, and molecular disorders not only in the liver but also in skeletal muscles, which, in turn, leads to impaired proteostasis [7].

The goal of treatment is to stop the progression of the disease and its complications, which requires a multidisciplinary approach, including nutritional support [1]. This review focuses on providing key dietary recommendations to patients with NAFLD, cirrhosis, and cholangiocarcinoma, as well as liver transplantation.

## 2. Nutritional Support for Liver Diseases

### 2.1. Cholangiocarcinoma

Cholangiocarcinoma is a known group of malignant cancers found in the biliary tree [8]. Notably, three types are highlighted: intrahepatic, perihilar, and distal [9]. Patients with cholangiocarcinoma are affected by poor prognosis. It is estimated that 5-year survival in the case of intrahepatic cholangiocarcinoma is around 10–49% [10]. Currently, surgery remains the most beneficial option of treatment. It is mentioned in this paper due to the fact that cholangiocarcinoma is a rare but aggressive tumor of the bile duct that is often diagnosed in its advanced stage with limited management [11]. Patients with bile duct cancers are at high risk of developing disease-related malnutrition; thus, they are also highly affected by both the primary and secondary consequences of malnutrition. Perioperative nutritional support is extremely important to prepare patients for surgical procedures and reduce the incidence of potential post-surgery complications, among other concerns. Noguchi et al. emphasized the importance of nutritional support prior to surgical treatment in the case of patients with intrahepatic cholangiocarcinoma [12]. In a study by Ma et al., the effects of early enteral nutrition (EEN) on the immune system were analyzed, as well as the clinical outcome of the patients with cholangiocarcinoma with malignant obstructive jaundice who underwent surgical treatment [13]. It was observed that EEN may improve the functioning of the immune system and the clinical outcomes of these patients [13].

#### 2.1.1. Oral Nutritional Supplements (ONS)

The administration of ONS provides beneficial effects in multiple conditions/diseases [14,15]. In the study by Kim et al., the effect of ONS consumption in patients with pancreatic and bile duct cancer who received chemotherapy was assessed [16]. The participants were divided into two groups: the first (*n* = 15) consumed ONS (at a dose of two packages per 8 weeks), and the second (*n* = 19) did not receive ONS. It was noted that the consumption of ONS significantly increased the daily intake of both energy and macronutrients, as well as positively affecting body mass. Moreover, it was observed that ONS alleviated fatigue symptoms [16]. The data on ONS usage in the case of bile duct cancer are still strongly limited. Nevertheless, there are some studies registered in the ClinicalTrials.gov system (such as “Influence of an Oral Nutritional Supplement Rich in Omega-3 Fatty Acids on Functional State and Quality of Life in Malnourished Patients with Gastroenterological Tumors” identifier: NCT00168987; diseases: colorectal neoplasm, hepatocellular carcinoma, cholangiocarcinoma).

#### 2.1.2. Omega-3 Fatty Acids

Omega-3 fatty acids are known to form part of immune nutrition, and they are characterized by anti-inflammatory properties [17]. Recently, it was also shown that omega-3 fatty acids support the production of butyrate in the human body because they increase the amount of butyrate-producing microbes, thus also modulating gut microbiota [18]. Interestingly, the deep molecular mechanisms in human cholangiocarcinoma cell lines (i.e., CCLP1 and TFK-1) were investigated in a study by Yao et al. [19]. It was observed that omega-3 polyunsaturated fatty acids induce the expression of 15-hydroxyprostaglandin dehydrogenase (15-PGDH) in cells by inhibiting the expression of miR-26a/b [19]. It should be emphasized that 15-PGDH is known as a key catabolic enzyme [20]. The effect of supplementation with omega-3 fatty acids in the case of patients with unresectable bile duct and pancreatic cancer who underwent chemotherapy was also investigated [21]. These patients received two to four packages of omega-3 fatty acids enteral nutrients (Racol^®^, Otsuka Pharmaceutical Factory, Tokyo, Japan) per day for 8 weeks. The dose of omega-3 fatty acids was 300 mg per package. It was observed that skeletal muscle mass increased after this nutritional support (at 4 weeks and 8 weeks after starting the administration of this product, *p* = 0.006 and *p* = 0.002, respectively). The authors concluded that supplementation with omega-3 fatty acids may improve the symptoms of cancer cachexia [21].

To sum up this part, it should be emphasized that the data regarding nutritional treatment in patients with bile duct cancers are still strongly limited. Nevertheless, it is deeply known that these patients are at high risk of malnutrition and the various consequences related to it. Perioperative nutritional support, enteral nutrition, ONS, and supplementation with omega-3 fatty acids seem to be promising strategies for patients with bile duct cancers.

### 2.2. Non-Alcoholic Fatty Liver Disease (NAFLD) and Non-Alcoholic Steatohepatitis (NASH)

Non-alcoholic fatty liver disease (NAFLD), first described in detail in 1980, is now one of the more common chronic liver diseases [22]. Progressive fatty liver is potentially reversible in its early stages, but if left undiagnosed and untreated, it can lead to non-alcoholic steatohepatitis (NASH) with advanced fibrosis (AF) and end-stage liver disease, which is an indication for liver transplantation [23]. It is estimated that the global prevalence of NAFLD can reach up to 25% of the entire population, and it is now the most common liver disease in Western countries [24]. The environment and lifestyle of Western countries favor the development of obesity and metabolic diseases. A highly processed, high-energy density, low nutrient density diet, a sedentary lifestyle, the easy availability of high-palatability food, and progressive industrialization are just some of the many environmental factors contributing to the occurrence of metabolic syndrome (MS) [25]. NAFLD is increasingly referred to as a hepatic manifestation of MS [26]; hence, the dietary and lifestyle intervention that works in MS also helps to reduce fatty liver in NAFLD patients [27].

It should be noted that in addition to overfeeding, some types of malnutrition may paradoxically promote the development of a fatty liver [28]. Hepatic steatosis has been observed in patients with kawashiorkor undernutrition, micronutrient deficiency undernutrition (iron, zinc, vitamin E, copper, and selenium), and subclinical undernutrition (environmental enteric dysfunction (EED)) [29].

The main dietary recommendations for MS include the principles of the Mediterranean diet, an energy deficit, and increasing physical activity during the day. These three components are the commonly accepted assumptions of an intervention supporting the treatment of metabolic syndrome [30]. However, there is still a population of NAFLD patients in whom the indicated intervention does not bring the desired results [31]. Moreover, a person with the lean-NAFLD phenotype cannot chronically use an energy deficit in the diet due to the risk of energy malnutrition [32]. A common feature of obese and lean NAFLD patients seems to be strong insulin resistance [27]. In some patients with NAFLD, disturbances in the intestinal microbiome, intestinal dysbiosis, endotoxemia, small intestinal bacterial overgrowth syndrome (SIBO), or intestinal barrier disorders (LGS) are observed [33,34,35]. The phenomenon of intestinal dysbiosis, disturbances in the quantitative and qualitative composition, as well as SIBO, may directly contribute to the unsealing of the intestinal barrier, bacterial translocation, or lipopolysaccharide (LPS) migration and the subsequent endotoxemia [35,36]. When taken together, this increases inflammation not only in situ, but also affects inflammation throughout the body [34,37,38]. This inflammation contributes to the development and worsening of insulin resistance, which creates a vicious cycle and disrupts the function of the liver [34]. Nutritional therapy in NAFLD should focus on improving the quantitative and qualitative composition of the gut microbiome, as well as on the normalization of its functions.

#### 2.2.1. Overall Recommendation

To date, one specific diet for NAFLD patients has yet to be identified. The 2019 European Society for Clinical Nutrition and Metabolism (ESPEN) [39] guidelines state that in overweight and obese NAFLD/NASH patients, 7–10% weight loss shall be aimed for to improve steatosis and liver biochemistry. Moreover, the grade B recommendation points out the Mediterranean diet as the recommended diet for NAFLD patients, which reduces steatosis and increases insulin sensitivity. There is a consensus that patients with NAFLD should increase their physical activity, mainly strength training (grade A recommendation; 100% consensus); this recommendation especially applies to people for whom weight reduction cannot be applied (lean-NAFLD) [39]. The nutrition guidelines for NAFLD patients are not consistent and specific. Although scientific associations, such as ESPEN, the Asian Pacific Association for the Study of the Liver (APASL), and the American Association for the Study of Liver Diseases (AASLD) [28], emphasize the importance of losing weight using an energy-restricted physical activity in reducing insulin resistance and general health, there are still no specified recommendations describing the type of diet and exercise [5,40,41]. Most scientific societies indicate the Mediterranean diet as the most beneficial model of nutrition for NAFLD patients, which has been confirmed by numerous scientific studies [5,42]. However, at the same time, there is currently a lack of evidence validating what macronutrient distribution of the diet is the most appropriate.

Additionally, salt intake should be limited already in the earliest stages of the disease for the purposes of the prevention of hypertension and cardiovascular diseases. In addition, a systematic review and meta-analysis of observational studies showed that populations with a high sodium intake have a 60% greater risk of developing NAFLD compared to those with low consumption, but the explanation of the mechanism of this phenomenon requires further research [43].

Interestingly, due to the presence of antioxidant compounds in coffee, such as caffeine, chlorogenic acid (CGA), cafestol, kahweol, and trigenolin, numerous studies on cell cultures, animal models, and humans have shown that coffee protects against metabolic syndrome and NAFLD/NASH. National Health and Nutrition Examination Surveys (NHANES 2001–2008) found that caffeine intake is independently associated with a lower risk of NAFLD [44].

#### 2.2.2. Mediterranean Diet

The Mediterranean diet consists mainly of plant-based ingredients: vegetable protein, plant polyphenols, and dietary fiber [45]. At least in part, it is these ingredients that may be responsible for the beneficial effects of the Mediterranean diet. Olive oil is the main source of fat in the Mediterranean diet, and its mono-unsaturated fatty acids, vitamin E, and antioxidants are associated with reduced cardiovascular and metabolic risks [46]. The fatty fish in the Mediterranean diet is a source of omega-3 fatty acids, which have anti-inflammatory and prebiotic effects [47]. In a randomized clinical trial, Meir A. Y et al. [48] compared the Mediterranean diet (MEDDiet) and the green Mediterranean diet (Green MEDDiet) in terms of the effect on steatosis in NAFLD patients. The GreenMEDDiet group consumed green tea (three–four cups/day), the Wolffia globosa aquatic plant strain (100 g/day frozen cubes), and a green shake with an additional portion of polyphenols (dose 1240 mg/day). Both the MEDDiet and reduced calorie Green-MEDDiet were shown to reduce body weight. Despite similar, moderate weight loss in both groups, the Green-MEDDiet group achieved a much greater loss of intra-hepatocyte fat (IHF%) (−38.9%) when compared to Mediterranean control (−19.6%; *p* = 0.035) and the general principles of the standard of care group (−12.2% proportional; *p* < 0.001). A greater loss of liver fat content was independently associated (*p* < 0.05) with an increase in the consumption of Wolffia globosa and walnuts, a decrease in the consumption of red and processed meat, and an increase in serum folate changes in microbiome composition (beta diversity) [48]. Another randomized trial by Pérez-Guisado J. et al. [49] tested the Mediterranean ketogenic diet on 14 obese men diagnosed with metabolic syndrome and NAFLD. At the end of the 12-week study, there was a significant decrease in alanine aminotransferase ALT (71.97 U/L vs. 37.07 U/L) and aspartate aminotransferase AST (47.71 U/L to 29.57 U/L). Additionally, there was a significant reduction in fatty liver in 92.86% of patients. Despite the small intervention group (*n* = 14), this study shows that the macronutrient composition of the diet may play a significant role in NAFLD diet therapy [49]. Studies on people with metabolic syndrome indicate some beneficial effects of intermittent fasting. Early time-restricted feeding (eTRF) is a protocol whereby there is a strictly defined period in the first quarter of the day when one eats and fasts [50]. The impact of eTRF was investigated in a study first conducted by Jamshed H. et al. [51] and all metabolic parameters (fasting glucose, fasting insulin, lipid profile, oxidative stress, hypertension, and postprandial insulin) were improved even without fat mass reduction [50,51]. Other studies (Xie, Z. et al., Sutton E.F. et al.) [50,52] also indicate the possible influence of the time of eating and the circadian rhythm on metabolic parameters and steatosis.

#### 2.2.3. Supplementation

**Figure 1:**
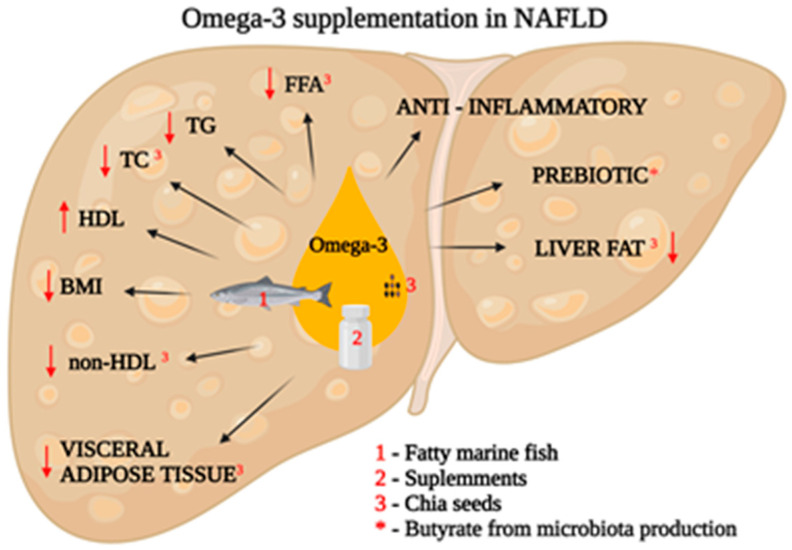
The effects of omega-3 fatty acids intake in NAFLD patients (BMI—body mass index; FFA—free fatty acids; HDL—high-density lipoprotein; non-HDL—non-high-density lipoprotein; TC—total cholesterol; TG—triacyloglicerydes. (Created with BioRender.com).

The role of supplementation in the nutrition of NAFLD patients is still not well understood. The ESPEN guidelines [39] point to the role of vitamin E supplementation (recommendation grade B) in patients with non-alcoholic steatohepatitis (NASH). However, there are no guidelines for NAFLD patients. In turn, supplementation with vitamin C, antioxidants (including resveratrol, anthocyanin, and coenzyme Q-10) or choline (no deficiency), omega-3, probiotics, and synbiotics is not recommended (zero recommendation, 100% consensus for all) [39]. However, it is worth noting that the lack of recommendations does not mean the potential effectiveness of the above-mentioned supplements; further research is required. A meta-analysis by Lee Ch. et al. [53] showed that omega-3 supplementation significantly reduced liver fat when compared to a placebo (pooled risk ratio 1.52; 95% confidence interval (CI) 1.09 to 2.13). Supplementation also significantly improved the triglyceride pool, total cholesterol, *High-Density Lipoprotein* (HDL), and body mass index (BMI), with a pooled mean difference and 95% CI of −28.57 (−40.81 to −16.33), −7.82 (−14.86 to −0.79), 3.55 (1.38 to 5.73), and −0.46 (−0.84 to −0.08), respectively. In a study conducted by Medina-Urrutia A. et al. [54], there was a significant improvement after dietary chia supplementation. Chia seeds (*Salvia hispanica*) are commonly known as a dietary source of omega-3 fatty acids. Medina-Urrutia A. et al. demonstrated that dietary chia supplementation decreases visceral adipose tissue (9%), body weight (1.4%), total cholesterol (2.5%), non-HDL (3.2%), and circulating free fatty acids (FFA) (8%) [54]. The effects of omega-3 fatty acids are presented in Figure 1.

Additionally, the researchers observed statistically significant (*p* < 0.05) NAFLD regression of over 52% in the intervention group. This effect may also be mediated by a much greater (over 55%) total dietary fiber consumption, as Chia is also a good source of dietary fiber [54]. Scorletti et al. investigated 1.0 × 109 CFU/d of probiotic strain Bifidobacterium animalis (subspecies lactis BB-12), combined with fructo-oligosaccharides (FOSs) with maltodextrin (8000 mg/day) [55]. Over 44–61 weeks in 104 NAFLD patients, the effect of the synbiotic supplementation vs. the control was not significantly different *p* = 0.30, and the between-group difference was 2.3% [55]. To date, there is no strong recommendation for dietary supplementation in NAFLD. Further studies are needed.

Many studies also indicated that some plant parts could have prebiotic and hepatoprotective properties: Table 1.

### 2.3. Cirrhosis

**Figure 2:**
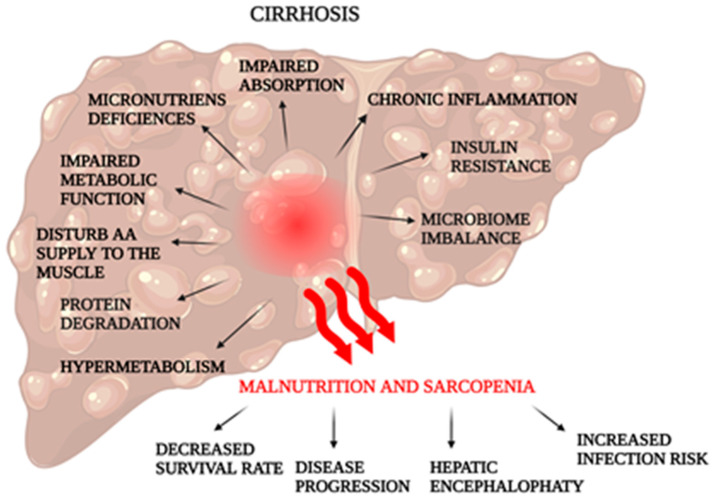
Causes and effects of malnutrition and sarcopenia in a patient with cirrhosis. (Created with BioRender.com).

Cirrhosis is characterized by severe fibrosis of the liver due to various factors, such as viral infection, drugs, and alcohol, as well as the end stage of chronic liver disease [4,92]. As a result of liver cirrhosis, absorption, and its functions, for example, metabolic, are impaired, such as the transformation of proteins, fat, carbohydrates, or toxic compounds, and as a result, cirrhosis becomes a systemic disease [4,93]. In addition, hypermetabolism is observed in 15–30% of patients with cirrhosis, and their resting energy expenditure is >120%, which adversely affects nutritional status [94,95,96]. During the progression to cirrhosis, the risk of malnutrition and sarcopenia increases—Figure 2 [2,97].

Sarcopenia is characterized by a decrease in skeletal muscle mass or strength and/or physical performance [98]. It is found in as many as 40–70% of patients with chronic liver diseases [99], and its etiology is usually multifactorial and includes chronic inflammation, insulin resistance, nutritional deficiencies, and endocrine disorders [100].

Loss of muscle mass may be a factor worsening the prognosis of patients with diseases of the hepatobiliary system. In the case of cirrhosis, there is talk of reduced survival and an increased risk of severe infection [99,101]. The loss of muscle mass in patients with cirrhosis is also associated with the development of hepatic encephalopathy odds ratio (OR) of 2.74 (95% CI, 1.87–4.01; I^2^ = 54.97%; *p* = 0.049) when compared to a population of patients with cirrhosis who do not have sarcopenia [102]. It is likely that muscle tissue provides an alternative pathway for ammonia detoxification (converts circulating ammonia to glutamine) [103]. When hepatic metabolism is impaired in advanced liver disease, ammonia accumulates in the serum. By penetrating the blood-brain barrier, it causes astrocyte edema, vasoconstriction, cerebral edema, and brain hypoperfusion [104].

In the case of the co-existence of sarcopenia with obesity, low muscle mass may be masked by excessive body weight, which increases the risk of cirrhosis decompensation and death in this group of patients [105,106].

The overall incidence of sarcopenia among patients with cirrhosis is 37.5% (95% CI 32.4–42.8%, *n* = 6.403), with a higher incidence among men than women (41.9%, 95% CI 35.8–48.2%, *n* = 3.141 vs. 28.7%, 95% CI 20.5–37.8%, *n* = 1.590, *p* = 0.012), patients with alcoholic liver disease when compared to non-alcoholic liver disease (49.6%, 95% CI 42.9–56.3%, *n* = 1.219 vs. 33.4%, 95% CI 27.4–39.6%, *n* = 2.166, *p* < 0.001), and greater severity of cirrhosis (Child-Pugh C) (46.7%, 95% CI 39.0–54.5%, *n* = 585). For comparison, patients with Child-Pugh class A saw 28.3%, 95% CI 20.5–36.8%, *n* = 1.143 (*p* = 0.007) [107].

Interestingly, patients with sarcopenia, compared to patients without sarcopenia, have an approximately 1.5-fold higher risk of NAFLD (OR 1.54 (95% CI, 1.05–2.26), with high heterogeneity between studies I^2^ = 83%, *p* < 0.0001) [108].

Sarcopenia is often associated with malnutrition [109]. In the population with cirrhosis, malnutrition is most often defined as a loss of skeletal muscle mass and/or strength and a decrease in subcutaneous and visceral adipose tissue mass. A common cause of these structural changes is a reduction in protein and energy intake [110]. In order to avoid underdiagnosis, malnutrition should be routinely screened in patients with cirrhosis. However, the interpretation of such a study may be biased by the effects of fluid retention, ascites, and peripheral edema [111].

Malnutrition can also be seen as a complication of cirrhosis as it has a negative impact on disease progression and outcome. Hospitalization and mortality rate are doubled in malnourished patients compared to adequately nourished patients, and malnutrition is an independent predictor of prognosis [112,113,114]. Malnutrition is also a predictor of the other complications of cirrhosis, in particular, infection and hepatic encephalopathy [115,116,117].

In advanced liver disease, both low muscle mass and obesity are important therapeutic targets that are modifiable through appropriate dietary interventions and/or exercise [118]. Such management has a positive effect on the prognosis of patients, such as improving nutritional status and reducing morbidity and mortality, even in patients with acute or chronic liver failure [93].

#### 2.3.1. Dietotherapy

So far, the effectiveness of various intervention therapies has been studied, which were parenteral nutrition (PN), enteral nutrition (EN), EN + intestinal probiotics, PN + EN, EN (without branched-chain amino acids BCAAs), late evening snack (LES), EN + LES, noLES. PN, EN, and PN+EN represent the routine therapy for cirrhosis, while EN+ intestinal probiotics, LES, and EN + LES have been at the cutting edge of therapy in recent years [119].

In general, the optimal diet for a patient with cirrhosis should be high in protein and high in energy due to the catabolic effects of cirrhosis and the associated increased protein degradation and, thus, loss of muscle mass [6,39]. As shown by Johnston et al. [118], a combination of dietary intervention (protein supply 1.2–2 g/kg/day) and physical activity (lasting ≥8 weeks, at least 3 days a week with 30–60 min of supervised aerobic and/or moderate-intensity resistance exercise) has the greatest potential for increasing muscle mass. In addition, physical training has a beneficial effect on skeletal muscles by reducing progression or even reversing muscle wasting [120] while improving physical fitness and fragility [121]. It is important that the intervention based on diet and physical activity is long-term [118].

The introduction of a late evening snack that is rich in carbohydrates (50 g of complex carbohydrates) shortens fasting time and eliminates the adverse changes resulting from night-time catabolism [122], as well as improving nitrogen metabolism and, in the long term, leads to an increase in lean body mass and reverses sarcopenia [123,124,125]. On the other hand, greater frequency, such as 5–6 meals a day, shortens catabolism episodes during the day [126].

In addition, for a complication called hepatic encephalopathy, it may be necessary to reduce the proportion of animal protein in favor of plant protein and dairy protein without affecting the overall protein content of the diet [6].

It should be noted that sodium intake should be restricted in patients in the advanced stages of the disease, especially in the case of ascites [5].

According to the ESPEN recommendations, parenteral nutrition (Grade GPP, strong consensus 100%) should be introduced in patients with moderate to severe cirrhosis who cannot eat food orally or cannot receive sufficient enteral nutrition. Additionally, parenteral nutrition should be given when fasting periods last longer than 72 h [5].

Interestingly, a meta-analysis of 16 studies (7 case-control studies and 9 cohort studies) involving patients with NAFLD or HCV and abusing alcohol showed that coffee consumption at any level (from low to high) reduces the risk of the development of liver fibrosis and cirrhosis compared to non-coffee drinkers (OR 0.73 and 0.61, respectively) [44].

#### 2.3.2. Supplementation

In addition, supplementation with branched-chain amino acids BCAA (Grade B, consensus 89%) has a positive effect on muscle mass [127,128]. In patients with cirrhosis, the supply of amino acids to the muscles is disturbed. BCAAs taken together or as single ingredients prevent lipolysis and proteolysis, stimulate albumin synthesis, improve nitrogen balance, have a positive effect on muscle mass, and also show improvements in hepatic encephalopathy, their general condition, and the quality of life in this group of patients [129,130]. BCAA, specifically leucine, lowers insulin resistance and is important in controlling glucose oxidation in skeletal muscle by stimulating the glucose-alanine cycle [122]. Plant protein can positively affect the composition of the intestinal microbiome [131].

The adequate supply of vitamins and minerals (Grade GPP, strong consensus 100%) should also be taken care of because their deficiencies are common due to the malabsorption of fats, the use of diuretics, and insufficient intake [132,133]. In addition, liver dysfunction itself can lead to changes in micronutrient metabolism. Reduced concentrations of zinc, selenium, iron, and magnesium are observed [134,135], while copper and manganese may be elevated [132,136]. Zinc supplementation may improve encephalopathy [5]. Deficiencies in fat-soluble vitamins are also common in cirrhosis due to the insufficient supply of bile acids [137]. Interestingly, prolonged vitamin D deficiency is associated with a reduction in type 2 muscle fibers [138]. On the other hand, vitamin D deficiency is also associated with an increased risk of NAFLD [139,140]. In addition, the level of water-soluble vitamins, especially vitamins C, B1, B2, B6, and folic acid, is lowered, whereas the level of vitamin B12 may be falsely elevated due to the release of vitamin B12 from damaged liver cells into the circulation [93].

In addition, supplementation with probiotics may, on the one hand, promote the growth of beneficial bacteria and, on the other hand, limit the growth of undesirable bacteria and the production of their harmful metabolites while supporting intestinal transport [141,142]. Based on the results of a meta-analysis and a network meta-analysis, Yu et al. [119] concluded that EN+ gut probiotics may be the most effective intervention, relatively speaking. In patients with cirrhosis, antibiotic therapy is often necessary, which leads to an imbalance of the intestinal flora, which results in the generation of enterotoxins and the metabolites of pathogenic microflora [119].

### 2.4. Liver Transplantation

The only available treatment for the end-stage of liver disease is transplantation. Patients with acute liver failure, end-stage chronic liver disease, primary liver cancer, or congenital metabolic disorders are eligible for surgery [143]. For most patients, transplantation is a life-saving and life-prolonging procedure [144]. In order for a patient to be qualified for surgery, they must meet a number of criteria, including being prepared for nutrition. The success of organ transplantation depends largely on the nutritional status of the patient. One of the primary problems in liver cirrhosis is malnutrition [145] caused by, for example, the following factors:

- A reduction in the amount of food consumed by the patient and an inadequate diet (i.a. low calorie diet low in protein (<0.8 g/kg bw) [146];
- metabolic process disorders (patients are characterized by a state of hypermetabolism; there is an acceleration of protein, fat and carbohydrate metabolism);
- the impaired absorption of fats associated with reduced bile secretion. This condition also leads to deficiencies in fat-soluble vitamins;
- the premature feeling of satiety (patients experience a delay in gastric emptying and ascites compressing the gastrointestinal tract) [93].

Due to the development of malnutrition as a result of liver cirrhosis, the risk of failure of the operation increases. Therefore, it is essential that each patient qualified for surgery undergoes a screening assessment of their nutritional status. If a state of emergency or malnutrition is found, it is necessary to treat it [147].

Among those patients qualified for liver transplantation, the risk of developing sarcopenia should be assessed, as this condition is a strong predictor of mortality and morbidity [39]. The assessment of the phase angle measured by bioelectrical impedance analysis is also mentioned as a prognostic factor of mortality among patients. In patients with cirrhosis, a low phase angle is associated with higher mortality [148]. Ways to control the adequate supply of protein using diet include anthropometric methods (e.g., measuring arm circumference) and biochemical methods (determining the level of albumin, prealbumin, total lymphocyte count (CLL), or nitrogen balance) [149].

#### 2.4.1. Nutritional Support before Liver Transplantation

Patients being prepared for surgery should maintain a diet dedicated to their current clinical condition. Depending on whether the patient has features of encephalopathy, the supply of protein in the diet should be modified. The goal of nutritional therapy before transplantation is to provide a diet with an optimal supply of energy and protein to counteract the development of malnutrition and to supplement the identified deficiencies of nutrients [145]. In hepatological patients, special attention should be paid to the possibility of deficiencies in fat-soluble vitamins, B vitamins, zinc, magnesium, and antioxidant micronutrients (selenium, vitamin E, and vitamin C) [150]. The recommendations in the preoperative and postoperative periods are presented in Table 2.

Patients at risk of malnutrition and meeting the following criteria: BMI < 18.5 kg/m^2^, weight loss > 10–15% within 6 months, and serum albumin < 30 g/L in the absence of liver or renal dysfunction are recommended to take oral nutritional supplements (ONS) for 7 days before the scheduled surgery date. In severely malnourished patients (loss of more than 10% WL), it is recommended to postpone surgery for 2 weeks. During this time, care should be taken to improve the patient’s nutritional status and increase the patient’s body weight [152].

It is recommended that patients prepared for liver transplantation should follow the guidelines of the Enhanced Recovery After Surgery (ERAS) protocol [147,151]. The guidelines indicate that in the preoperative period, the caloric content of the diet should be 30–35 kcal/kg/d, and the protein supply should be at the level of 1.5 g/kg/d, following the standard nutrition scheme (Evidence level: high; Grade of recommendation: strong) [151].

The effect of BCAA supplementation in patients undergoing liver transplantation was evaluated in the studies of Kaido et al. [157] and Shirabe et al. [158]. The studies indicated a significant improvement in the treatment of sepsis [157] and a reduction in bacteremia in the postoperative period [159]. It should also be noted that the current rules of ERAS protocol do not indicate the need to include such preparations in patients [147].

There is evidence to include probiotics before or on the day of liver transplantation. Depending on the study, the duration of supplementation was different, and a variable number of strains was also used (Evidence level: high; Grade of recommendation: weak) [151,159,160]. The meta-analysis conducted by Sawas et al. [161] also indicates the effectiveness of administering a combination of prebiotics and probiotics to patients before or on the day of transplantation, reducing the frequency of perioperative infections or the duration of prescribed antibiotic therapy [161]. At the same time, the ERAS protocol does not indicate exact recommendations in this area [147].

In the case of preoperative immuno-nutrition (IN), there is no clear evidence to support its use; therefore, IN before liver transplantation is not recommended (evidence level: high; grade of recommendation: weak) [147,151]. At the same time, there is a study showing that the use of preparations enriched with arginine, omega-3 fatty acids, and nucleotides before the procedure has a positive effect on reducing the frequency of infectious complications and shortening the length of stay after operations in the area of the gastrointestinal tract [153].

Patients preparing for surgery should be careful about the use of any herbs because the compounds contained in them may interact with any medications taken and have hepatotoxic effects [162].

##### Before the Surgery—ERAS Protocol

According to the ERAS procedure, the patient’s fasting period should be minimized, and the use of protein restrictions in the case of HE is abandoned (recommendation grade GPP, strong consensus 100%) [5]. In patients with hepatic insufficiency, hepatic glycogen is depleted; therefore, it is important to shorten the fasting period of the patient as much as possible. There is no indication that preoperative fasting should exceed 6 h for solid food and 2 h for fluid intake. Thanks to this, the body will not start the process of muscle protein breakdown as a result of ongoing gluconeogenesis and will not deepen the development of sarcopenia. If there are risk factors for late gastric emptying, such as diabetes mellitus, tense ascites, or autonomic dysfunction, care should be taken in preparing the patient [151].

The ERAS protocol assumes that the patient should drink a clear carbohydrate fluid 2 h before surgery. The available literature indicates a beneficial effect of carbohydrate loading before liver surgery on the reduction of postoperative insulin resistance [154]. The beneficial effects of a high-carbohydrate drink were also confirmed in a study by Plank et al. In patients with end-stage liver failure, the administration of the carbohydrate drink helped to improve preoperative nutritional status, reduce infectious complications, and shorten the time of recovery after transplantation. At the same time, the authors of the study noted that the benefits they indicated should be confirmed in a randomized control study [160].

##### Limitations of Implementing the ERAS

The ERAS protocol assumes the introduction of ONS 7 days before the operation. However, in the case of transplantation, information about the possibility of surgery often comes at the last minute. The situation is similar to the use of the full carbohydrate-rich drink procedure to reduce the preoperative fasting time. For patients who are severely malnourished and matched with the donor, it is not possible to postpone the operation by 2 weeks [147,152].

Although the ERAS pathway for liver transplantation is based on the best available evidence, it still requires further research [151].

#### 2.4.2. Nutritional Support after Liver Transplantation

Patients undergoing liver transplantation have similar caloric needs to those after abdominal surgeries [5]. Feeding should be started as soon as possible after the operation is completed. This approach reduces the risk of postoperative symptoms such as nausea, hunger, thirst, and malaise [154].

##### Early Period (12–24 h)

According to the ERAS protocol, normal oral food intake and/or enteral nutrition (nasogastric tube/jejunostomy) should be started within 12–24 h after the surgery (depending on patient tolerance). Additional nutritional support should be provided to malnourished patients and patients undergoing prolonged postoperative fasting lasting more than 5 days, e.g., in the event of complications [147,155]. The inclusion of parenteral nutrition is considered in a situation where it is not possible to use the oral route (jejunostomy/enteral tube).

Due to the lack of clear evidence, there is no obligation to use dietary supplements in patients after liver transplantation [147]. It should be noted that a high percentage of patients use herbal preparations and dietary supplements; therefore, they should be educated about possible toxicity and drug interactions [163].

##### Later Period

People who have undergone a liver transplant have a higher risk of developing diabetes due to the immunosuppressive drugs used. If diagnosed, the patient should modify their diet by, e.g., limiting the consumption of simple sugars. The hyperglycemic effect of tacrolimus can be reduced by reducing the dose [150].

Immuno-suppressive drugs can also lead to increased potassium levels. If such a situation occurs, it is recommended that the patient limit the products of its source in the diet. This situation usually occurs in the early perioperative period [156]. Among the patients, a reduced level of magnesium can also be observed. In order to increase its level, it is recommended to use a balanced diet rich in, for example, whole grains, groats, legumes, or nuts. Taking magnesium supplements can lead to diarrhea [150].

Liver transplantation is the main definitive treatment for end-stage liver disease and eliminates several factors contributing to the pathogenesis of sarcopenia. Hepatocyte function is restored, ascites regresses, and portal pressure is lowered. On the other hand, the patient must take immunosuppressive drugs that adversely affect muscle mass by activating myokines, as well as increasing fat mass (sarcopenic obesity). Studies have shown that muscle mass can stabilize, increase, or decrease after a liver transplant. So, there are no clear results on this. Post-transplant obesity, in addition to the effect of drugs, may also be partly related to excessive caloric intake. Thus, the patient’s adherence to a nutritional plan with an adequate supply of protein and calories may reduce the risk of developing obesity, insulin resistance, and sarcopenic obesity [7].

The largest increase in body weight usually occurs in the first 6 months after the operation [155]. It should be noted that the increase in body weight is usually continuous, and patients also gain weight in subsequent years, which results in the development of obesity and being overweight [150].

## 3. Future Perspectives and Limitations

This review focuses on presenting the key nutritional recommendations in the successive stages of chronic liver disease. Unfortunately, they are not always possible to implement in clinical practice, e.g., in the case of preparing a patient for liver transplantation, due to the unpredictability of this procedure. These recommendations may also be too difficult for the patient to follow on their own. In addition, the above recommendations must be introduced at an early stage of the disease in order to have the desired effect. This is problematic because even cirrhosis and liver cancer can develop asymptomatically, and the symptoms are felt by the patient only in the late stages of the disease when treatment is already very difficult. These suggest that the use of general dietary recommendations may be limited.

The manuscript does not present detailed recommendations regarding physical activity and its therapeutic significance in various stages of the disease, although, as a factor stimulating anabolism, it is an indispensable and helpful element of therapy.

The challenge for future research is to develop tools to support patients in meeting dietary recommendations in a more individualized way, as well as to facilitate the control of compliance with these recommendations. In such a personalized approach, it is important to take into account biochemical, anthropometric, microbiota, and lifestyle parameters.

## Figures and Tables

**Table 1:** Examples of plants with prebiotic and hepatoprotective properties.

Common Name (Latin Name)	Family	Used Part	Materials	References
Garlic (*Allium sativum*)	*Alliaceae*	bulb	Rats	[56,57,58]
Onion (*Allium cepa*)	*Alliaceae*	bulb	Rats	[57,59,60,61,62]
Artichoke (*Cynara scolymus*)	*Apiaceae*	leaves, flowers	Rats, clinical trials (100 patients, 17 patients)	[56,57,59,63,64]
Dandelion (*Taraxacum officinale*)	*Asteraceae*	root, leaves	Mice	[56,57,64,65,66,67]
Chicory (*Cichorium intybus*)	*Asteraceae*	root, herb	Rats, mice, rabbits, clinical trials (92 patients, 36 patients)	[59,63,64,68]
Reishi or Lingzhi (*Ganoderma lucidum*)	*Polyporaceae*	fruiting bodies	Mice, rats,	[69,70,71,72,73]
Antrodia cinnamomea *(syn. A. camphorata*)	*Fomitopsidaceae*	fruiting bodies	Rats, mice, female chicks, human HCC cell lines (i.e., HepG2 and PLC/PRF/5), clinical trials (44 patients, 28 patiensts)	[69,74,75,76,77,78,79,80]
Mushroom (*Agaricus bisporus*)	*Agaricaceae*	fruiting bodies	Mice	[81,82,83,84,85]
Golden oyster mushroom (*Pleurotus citrinopileatus*)	*Pleurotaceae*	fruiting bodies	Mice, Human cell line (HepG2 cells)	[86,87,88]
Shiitake (*Lentinus edodoes*)	*Marasmiaceae*	fruiting bodies	Mice	[87,89,90,91]

**Table 2:** Recommendation in the pre-operative and postoperative period.

Symptoms/Patient’s Condition		Recommendation
PREOPERATIVE PERIOD		
	Current condition of the patient	Diet adapted to the patient’s condition [145];
		optimal supply of energy: 30–35 kcal/kg BW/day [151]; obese patient: EN or PN 25 kcal/kg IBW/day [151]
		optimal supply of protein:1.2–1.5 g/kg) [151]; obese patients: 2.0–2.5 g/kg IBW/day [151];
		optimal supply of fat-soluble vitamins, B vitamins, zinc, magnesium, antioxidants (selenium, vit E, and vit C) [150];
	Hepatic encephalopathy	Modified protein intake [145];
	Malnourished patients (BMI < 18.5 kg/m^2^; weight loss > 10–15% within 6 months; serum albumin < 30 g/L)	Oral nutritional supplements (ONS) for 7 days before surgery [152];
	Severely malnourished patients (more than 10% WL)	Surgery postponed by 2 weeks [152];
		Improving nutritional status [152];
	Infectious complications risk	Formulas enriched with arginine, omega-3 fatty acids, nucleotides [153];
	Preoperative fasting	Shortening fasting period [151];
		6 h for solid food and 2 h for fluid before surgery [151];
	Postoperative insulin resistance	Clear carbohydrate fluid 2 h before surgery [151,154];
	POSTOPERATIVE PERIOD	
Early period 12–24 h		Oral food intake and/or enteral nutrition within 12–24 h after surgery (30–35 kcal/kg/day; protein: 1.2–1.5 g/kg/day) [147,155];
	Malnourished patients OR prolonged postoperative fasting (>5 days)	Additional nutritional support [147,155];
	Oral route impossible	Parenteral nutrition [147];
Later period	DM risk (immunosuppressive drugs)	Reduce consumption of simple sugars,
		reduce Tacrolimus dose [150];
	Increased potassium level (immunosuppressive drugs)	Limit the food products rich in potassium [156];
	Decreased magnesium level	Balanced diet (whole grains, legumes, nuts) [150];
	Excessive body weight (overweight, obesity)	Balanced diet, proper protein intake [150];

BW—body weight; DM—Diabetes mellitus; EN—enteral nutrition; IBW—ideal body weight; PN—parenteral nutrition; WL—weight loss.

## Data Availability

Data sharing not applicable No new data were created or analyzed in this study. Data sharing is not applicable to this article.
